# Deciphering the role of CA1 inhibitory circuits in sharp wave-ripple complexes

**DOI:** 10.3389/fnsys.2013.00013

**Published:** 2013-05-06

**Authors:** Vassilis Cutsuridis, Jiannis Taxidis

**Affiliations:** ^1^Institute of Molecular Biology and Biotechnology, Foundation for Research and Technology - HellasHeraklion, Greece; ^2^Computation and Neural Systems, California Institute of TechnologyPasadena, California, CA, USA

**Keywords:** sharp wave-ripple complexes, inhibition, hippocampus, memory consolidation, medial septum

## Abstract

Sharp wave-ripples (SWRs) are population oscillatory patterns in hippocampal LFPs during deep sleep and immobility, involved in the replay of memories acquired during wakefulness. SWRs have been extensively studied, but their exact generation mechanism is still unknown. A computational model has suggested that fast perisomatic inhibition may generate the high frequency ripples (~200 Hz). Another model showed how replay of memories can be controlled by various classes of inhibitory interneurons targeting specific parts of pyramidal cells (PC) and firing at particular SWR phases. Optogenetic studies revealed new roles for interneuronal classes and rich dynamic interplays between them, shedding new light in their potential role in SWRs. Here, we integrate these findings in a conceptual model of how dendritic and somatic inhibition may collectively contribute to the SWR generation. We suggest that sharp wave excitation and basket cell (BC) recurrent inhibition synchronises BC spiking in ripple frequencies. This rhythm is imposed on bistratified cells which prevent pyramidal bursting. Axo-axonic and stratum lacunosum/moleculare interneurons are silenced by inhibitory inputs originating in the medial septum. PCs receiving rippling inhibition in both dendritic and perisomatic areas and excitation in their apical dendrites, exhibit sparse ripple phase-locked spiking.

In recent years a wealth of knowledge about the anatomical, physiological, molecular, and synaptic properties of the various cell types in the hippocampus has accumulated. Apart from the numerous different identified classes of hippocampal interneurons targeting specific parts of pyramidal cells (PC) (Freund and Buzsáki, 1996; Klausberger and Somogyi, 2008) and a complex set of intra- and extra-hippocampal excitatory inputs (Witter, 2010) there is also increasing evidence on the important role of inhibition between interneurons (Chamberland and Topolnik, 2012) in sculpting the activity of PCs. However, the interaction mechanisms of such complex circuitries during network oscillations, either extrahippocampally paced or internally generated (Buzsaki, 1989; Cobb et al., 1995), still remain elusive. Particularly, during high-frequency oscillatory events, like the SWR complex.

SWRs are primary hippocampal activity patterns, observed in local field potentials (LFPs) from rodents, primates and humans, during deep sleep, anaesthesia, and awake immobility. They are observed synchronously throughout the hippocampus (Chrobak and Buzsáki, 1996) and have a typical duration of 30–120 ms, recurring at ~1 Hz. SWRs are generated by strong depolarizing inputs from CA3 population bursts, exciting CA1 cells through the Schaffer collaterals (Buzsáki et al., 1992; Ylinen et al., 1995; Csicsvari et al., 2000). They consist of a sharp depolarization in the CA1 dendritic layer (sharp wave), accompanied by transient oscillatory LFP patterns of ~150–200 Hz (ripple) located in the CA1 pyramidal layer (Ylinen et al., 1995). During SWRs, ensembles of place cells replay in faster timescale their sequential activity, acquired during awake exploration (Skaggs and McNaughton, 1996; Foster and Wilson, 2006; Diba and Buzsáki, 2007; Dragoi and Tonegawa, 2011). Such fast-scale replays, along with their correlation with neocortical activity (Peyrache, 2009) and the memory-impairment observed during ripple-disruption (Girardeau et al., 2009) suggest a crucial role for SWRs in memory consolidation.

As an intrinsic CA1 oscillation, ripples are generated by the rich anatomical and functional connectivity within CA1. PCs receive inputs in their distal dendrites from layer III of the entorhinal cortex (EC), through the perforant path, and in their proximal dendrites from the CA3 Schaffer collaterals, as a portion of the trisynaptic loop. PC axons target mainly subicular and neocortical areas, and recurrent excitation is very low (less than 1%) in CA1 (Amaral and Witter, 1989). In addition to excitatory cells, at least 21 different types of inhibitory interneurons have now been identified in regions CA1 and CA3 (Freund and Buzsáki, 1996; Somogyi and Klausberger, 2005; Fuentealba et al., 2008a,b; Cutsuridis et al., 2010a,b; Capogna, 2011). These cells are distinguished based on their anatomical, morphological, pharmacological, and physiological properties. They include the axo-axonic cells (AAC), the perisomatic-targeting basket cells (BCs) and the dendritic-targeting bistratified (BSC), ivy (IVY), neurogliaform (NGL) and oriens lacunosum-moleculare (OLM) cells (Freund and Buzsáki, 1996; Fuentealba et al., 2008a,b; Klausberger and Somogyi, 2008; Capogna, 2011). AACs are fast spiking interneurons innervating exclusively the initial axonal segment of the PCs, whereas BCs innervate their cell bodies and proximal dendrites (Klausberger et al., 2003). BSCs and IVYs innervate the PC basal and oblique dendrites, whereas OLM and NGL cells target the apical dendritic tuft of PCs aligned with the EC input (Klausberger et al., 2003, 2004). AACs and BCs receive excitatory inputs from both the EC and the CA3 Schaffer collaterals, whereas BSCs receive inputs only from CA3 and NGLs only from the EC (Klausberger and Somogyi, 2008; Capogna, 2011). IVYs and OLMs are recurrently excited by CA1 PCs (Fuentealba et al., 2008a,b; Klausberger and Somogyi, 2008).

The different CA1 excitatory and inhibitory neurons display diverse firing patterns during SWRs (Klausberger et al., 2003, 2004; Fuentealba et al., 2008a,b; Klausberger and Somogyi, 2008; Royer et al., 2012). Experimental studies have shown that during an SWR episode, AACs fire first, followed by BSCs, followed by PCs and BCs [Figure 2 in Klausberger and Somogyi (2008)]. Specifically, AACs fire just before the onset of the ripple episode, whereas PCs, BCs, and BSCs fire in phase with the ripple (Ylinen et al., 1995; Klausberger and Somogyi, 2008). OLMs are silent during the fast ripple (Klausberger and Somogyi, 2008), firing only toward the end of the SWR (Pangalos et al., 2013). Similarly, medial septal (MS) GABAergic neurons, which target hippocampal inhibitory interneurons (Freund and Antal, 1988), differentially phase their activities with respect to SWRs (Dragoi et al., 1999). Some MS GABAergic cells pause their activities just before the peak of the ripple and increase their firing right after it (type 1A), whereas others pause their activities during the entire duration of the ripple episode (type 1) (Dragoi et al., 1999). Understanding how these different types of CA1 and MS excitatory and inhibitory cells contribute to the generation of SWRs is of great importance because of the crucial role of SWRs on memory consolidation through the compressed replay (forward and reverse) of memories acquired during wakefuleness. Yet, the actual mechanisms that control spiking activity, giving rise to the fast ripple oscillations, while allowing PCs to fire at particular temporal windows during the ripple oscillation (Klausberger and Somogyi, 2008) are still unknown.

Early theoretical studies (Traub and Bibbig, 2000) predicted that axon-axon gap junctions between PCs in networks of PC and somatic inhibitory interneurons coupled with chemical synapses can generate coherent population oscillations at frequencies greater than 100 Hz. But recent experimental studies (Ellender et al., 2010) showed that tight control of excitation and GABA-A mediated fast feedforward perisomatic inhibition is sufficient for the generation of SWRs in the hippocampal slice.

Based on the observations that inhibition is necessary for SWR generation (Ellender et al., 2010) and that BCs dramatically increase their firing during SWRs (Klausberger and Somogyi, 2008), firing in phase with ripples (Ylinen et al., 1995; Csicsvari et al., 1999), a recent neural network computational model (Taxidis et al., 2012, 2013) reproduced basic LFP ripple characteristics proposing a perisomatic inhibition-based mechanism for SWR generation. The model consisted of a CA3 and a CA1 network, both one dimensional arrays of two-compartment (dendritic and axosomatic) PCs and single-compartment fast-spiking perisomatic interneurons, interconnected in a simplified but realistic topology. CA3 was characterized by an extensive recurrent excitatory network, while strong fast-decaying, recurrent inhibition underlay CA1 topology. CA3 drove CA1 PCs and interneurons through a set of excitatory connections, mimicking Schaffer collaterals. The strength of the Schaffer drive was uniform for interneurons, but varied throughout the pyramidal population, creating a “strongly-driven subset” of cells. LFPs were modeled as summed local synaptic conductances. Pyramidal spiking combined with the recurrent excitation of the CA3 model produced population bursts quasi-synchronized over the whole CA3 network and regulated by feedback inhibition. These bursts excited CA1 interneurons which, through their local recurrent inhibition, quickly synchronized their spiking in ripple-frequency oscillations (~150–200 Hz). PCs received the excitatory Schaffer-drive in their dendritic compartment, giving rise to a sharp-wave LFP, along with the oscillating inhibition in their somatic compartment, closely resembling ripple LFPs. Only the strongly-driven pyramidal subset overcame inhibition and produced spikes that closely preceded the interneuronal spike cycle and were phase locked to the ripple troughs, in accordance with electrophysiological observations (Ylinen et al., 1995; Csicsvari et al., 1999).

Nevertheless, the model does not address the variable roles of the different identified classes of hippocampal interneurons targeting specific parts of PCs. Cutsuridis and Hasselmo (2011) were the first that attempted to address such issues from a computational perspective: (1) How are storage and replay (forward and reverse) of temporally ordered memory patterns controlled by the CA1 microcircuit during theta oscillations and SWRs? (2) What roles do the various types of inhibitory interneurons play in these processes? To this end, they formulated a canonical network model of four PCs and four types of inhibitory interneurons: AAC, BC, BSC, and OLM cells. The model simulated accurately the firing of different hippocampal and MS cell types relative to theta oscillations and SWRs in urethane-anaesthetized rats (Dragoi et al., 1999; Klausberger and Somogyi, 2008). In accordance to experimental evidence, the model proposed that in the case of SWRs, when a CA3 highly synchronous activity (not modeled) drove the model's CA1 PCs and interneurons, the activities of the CA1 and MS interneurons were sculpted by their mutual inhibition (Freund and Antal, 1988). The AAC activity was halted by the rhythmic inhibition of the MS type 1A cell (Dragoi et al., 1999), whereas the BC and BSC were disinhibited by the MS type 1 cell (Dragoi et al., 1999) which has been shown to pause its activity during the entire SWR episode (Dragoi et al., 1999). The role of the AAC in the model was to silence the CA1 network and prepare it for the appropriate replay of information based on the current context. BCs' role was to hyper-synchronize the PCs activities and make them fire at ripple frequency (>100 Hz), whereas BSCs' role was to provide an inhibitory threshold mechanism to all PCs in the network, allowing only the correct in order PC to replay the memory. The OLM cell was silent during the SWR episode (Klausberger and Somogyi, 2008). Despite the model's success in reproducing the cells' responses to SWRs, it did not address the mechanism of SWR generation.

Moreover, recent experimental reports, all based on combined optogenetic, juxtacellular, and pharmacological approaches, shed new light on the role of various interneuronal classes in shaping the CA1 spiking output (Lapray et al., 2012; Leão et al., 2012; Lovett-Barron et al., 2012; Royer et al., 2012; Pangalos et al., 2013). By driving Cre expression with either PV- or SOM-expressing interneurons in CA1 slices, Lovett-Barron et al. (2012) showed that SOM interneurons (mainly dendritic BSCs), can modulate pyramidal spiking output from Schaffer collateral stimulation, more efficiently than PV-interneurons (mainly perisomatic BCs), by controlling dendritic electrogenesis. Silencing BSCs allowed an NMDA-driven generation of dendritic spikes that turned PCs from regular spikers to bursters. Similar results were reported *in vivo* by Royer et al. (2012), on mice running on a treadmill belt, who also revealed a role for BCs in controlling not the pyramidal output but rather the timing of pyramidal spikes, particularly place cell spiking relative to the theta phase. By identifying an OLM-specific molecular marker, Leão et al. (2012) produced transgenic mice were they optogenetically silenced OLM interneurons. This technique revealed a role for these cells in controlling (suppressing) the influence of the entorhinal input on pyramidal distal dendrites, while enhancing the influence of the Schaffer collateral input on apical dendrites, possibly by inhibiting SOM dendritic interneurons. Finally, Lovett-Barron et al. (2012) also showed that BCs can effectively inhibit BSCs and (more weakly) OLM cells, providing an additional, indirect control for dendritic spike generation and pyramidal output. When combined, these three studies draw the picture of a rich and intriguing interplay between distal dendrite-, proximal dendrite- and perisomatic-targeting interneurons in shaping the pyramidal spike output during various stimulation protocols. The way this interplay functions during SWRs, shaping the spiking output of PC, is still unknown.

We attempt to incorporate these new findings in a conceptual model on how various forms of somatic and dendritic inhibition may collectively contribute to the generation and maintenance of SWRs in region CA1, while at the same time providing functional roles for the various CA1 and MS cells during SWRs (Figure 1). In our conceptual model, SWRs in CA1 are generated as in the Taxidis et al. (2012) model: CA3 PC spiking combined with their strong recurrent excitation produces population bursts, which are quasi-synchronized over the whole CA3 network and regulated by feedback inhibition. These CA3 bursts then excite the CA1 PCs along with classes of INs that have dendritic arborizations in stratum radiatum and/or oriens, mainly AACs, BSCs and BCs.

**Figure 1:**
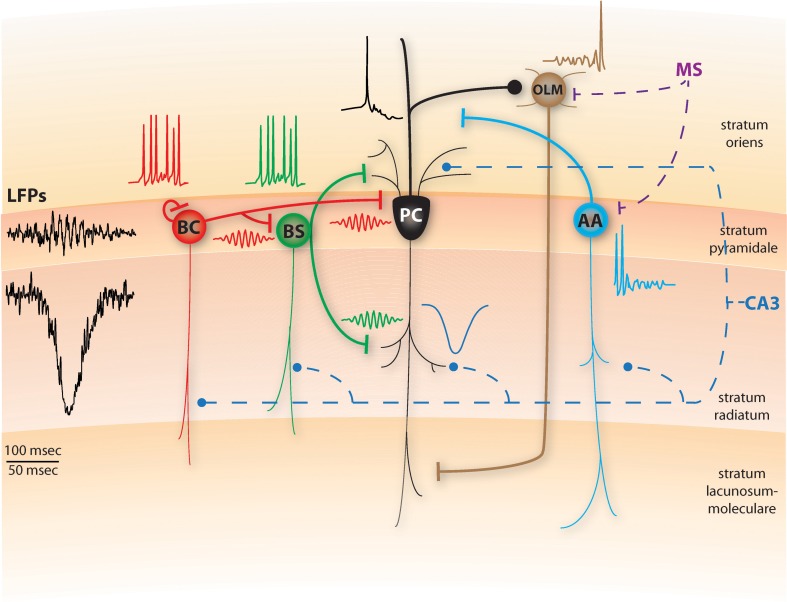
**Schematic diagram of a conceptual SWR model, including pyramidal cells (PC), basket cells (BC), bistratified cells (BS), axo-axonic cells (AAC) and oriens-lacunosum moleculare cells (OLM).** Thin lines represent dendritic arborizations and thick lines are axonal ones. Dashed lines represent inputs arriving from CA3 through the Schaffer collaterals (blue) or the medial septum (MS, purple). The representative spiking activity of BS, BC and PC cells during SWRs is shown above the corresponding cells. In the model, the BS cells will respond to the CA3 input by fast spiking which abolishes dendritic electrogenesis and somatic bursting in the PCs. BC cells also spike fast and due to their recurrent inhibition, synchronize their firing in ripple frequencies, imposing this rhythm on the BS population as well (red and green ripple-frequency spikes). Hence, PCs receive synchronous ripple-frequency inhibitory inputs in their dendritic and perisomatic areas (green and red ripples) along with excitatory input in their apical dendrites (blue, sharp-wave), yielding ripple-frequency intracellular oscillations and ripple phase-locked sparse firing (black trace). A schematic SWR LFP that would result from this activity is shown on left. AA cells also received the CA3 excitation, but respond only during the initial stages of SWRs as MS inhibition later dominates (cyan trace). Finally, the OLM cell remains silent throughout the main SWR event, due to the MS inhibition and the lack of any CA3 excitation, only being able to spike at later stages of SWRs when the excitation from CA1 cells has built up (brown trace). Top timescale corresponds to LFPs; bottom one to all spiking and synaptic traces. All traces are conceptual and start at the same timepoint.

During the onset of the CA3 population burst, AACs are the first to respond by increasing their spiking (Klausberger et al., 2003). Yet, the combined rhythmic inhibition in their basal dendrites, stemming from the MS type 1A inhibitory cells (Dragoi et al., 1999), pauses their activity during the SWR and immediately after it (Klausberger and Somogyi, 2008). As in Cutsuridis and Hasselmo (2011), the role of AACs in our model is to silence the pre-SWR PC spiking output to prepare the network for the upcoming replay of information based on the current context (Figure 1). Their silencing during SWRs disinhibits the PC axons, promoting the transfer of spike patterns to their neocortical targets.

BSCs are the second interneuronal class to respond to the strong CA3 excitation (Klausberger et al., 2004), inhibiting the PC basal and oblique dendrites. In light of the recent experimental evidence (Lovett-Barron et al., 2012), we suggest that the functional role of the BSC inhibition is to control the firing rate of CA1 PCs, turning them from bursters to regular spikers, by blocking dendritic NMDAR-dependent spikes, thus abolishing somatic bursting. As a result, blocking NMDA pharmacologically has no significant effect on SWRs (Ellender et al., 2010) and slow Ca^2+^ dendritic spikes are rarely observed during SWRs (Kamondi et al., 1998).

BCs are the third interneuronal class to respond to the CA3 population bursts, increasing their firing slightly after BSCs (Klausberger et al., 2004). Following the Taxidis et al. (2012) network model, we suggest that the local fast-decaying recurrent inhibition between BCs quickly synchronizes their spiking in ripple-frequency oscillations (Figure 1). Since BCs can also effectively inhibit BSCs (Lovett-Barron et al., 2012), we suggest that the BC rhythmic inhibitory output synchronizes the BSC population as well, in ripple-modulated spiking that is in phase with the BCs, following the LFP ripple troughs by 1–2 ms (Klausberger et al., 2004).

As a result, from the first stages of the SWR on, CA1 PCs receive a barrage of excitatory inputs throughout their apical and basal dendrites via Schaffer collaterals, combined with a ripple-frequency oscillating inhibition that is synchronous throughout their dendritic arborization and their soma, reflected in intracellular voltage oscillations (Figure 1, Ylinen et al., 1995). In accordance with recent evidence that BCs control the fine timing of pyramidal spikes during theta (Royer et al., 2012), we propose that the rhythmic inhibition PCs receive during SWRs limits their spiking output in narrow time windows formed by the peaks of inhibition. Pyramidal spikes can mostly occur few milliseconds after the maximal inhibition has decayed and before the next inhibitory peak, resulting in spike histogram peaks that slightly precede BC/BSC peaks, phase locked with ripple troughs (Ylinen et al., 1995; Csicsvari et al., 1999). Moreover, only the most strongly Schaffer-driven PCs will overcome the inhibition and produce spikes (Taxidis et al., 2012, 2013). Since PC spiking is relatively sparse on the individual cell level (Ylinen et al., 1995), the feedback excitation from PCs to BCs will have a minimal role relative to the massive feedforward input from CA3. Hence, BCs' role during SWRs is to hyper-synchronize the PC firing in ripple-periodic temporal windows (Ellender et al., 2010; Cutsuridis and Hasselmo, 2011; Taxidis et al., 2012). Finally we hypothesize that the temporal sequence in which place-encoding PCs spike during the SWR is controlled by the Schaffer-input that stems from corresponding replay in CA3 PCs (Cutsuridis and Hasselmo, 2011).

Although BC axons have been shown to make synaptic contacts to cells located in stratum oriens (e.g., OLM cells, Klausberger et al., 2003), the BC inhibition to OLMs appears to be too weak (Lovett-Barron et al., 2012). In our conceptual model, during the peak of the SWR episode, OLM cells are strongly inhibited by the rhythmic type 1A MS inhibitory cells (Dragoi et al., 1999), which can overpower the PC regular spiking excitation they receive (Pangalos et al., 2013), silencing most of them (Klausberger and Somogyi, 2008; Cutsuridis and Hasselmo, 2011), thus disinhibiting BSCs (Leão et al., 2012). Only toward later stages of the SWR, the excitation received by the pyramidal output allows OLM cells to spike (Figure 1) (Pangalos et al., 2013).

This theoretical model combines the computational approaches of Cutsuridis and Hasselmo (2011) and Taxidis et al. (2012), suggesting both a generation mechanism for ripple oscillations and a functional role for some basic CA1 interneuronal classes during SWRs. It also incorporates the recent experimental observations on the role of dendritic and somatic inhibition in CA1, expanding them in the SWR framework. A number of outstanding questions arise from our conceptual model:

- What functions do CA1 PCs serve when they produce bursts as opposed to when they fire regular spikes? If PCs would turn to busters by silencing of BSCs during SWRs, what would the effect be on the fidelity of pattern replays and consequently on memory-task performance?
- What is the functional role of the AAC turn-off on the transfer of the PC output to its synaptic targets?
- How is the activity of MS GABAergic cells controlled by SWRs and what is its functional role in CA1? How would the silencing of type 1A MS cells, during SWRs, affect their CA1 interneuronal targets and consequently SWRs?
- What effect does the BSC inhibition have in synaptic plasticity properties of CA1 PC thin oblique dendrites during SWRs?
- Assuming that spike sequence replays are generated within CA3, what are the exact synaptic/network mechanisms within CA1 controlling their transfer to extrahippocampal targets?
- What is the functional role of the plethora of other interneuronal classes during SWRs, not addressed here?
- Which intrinsic cell properties and network features need to be incorporated in a computational model to simulate the characteristics of our conceptual framework? What would such a computational model predict about CA1 functionalities in non-SWR hippocampal states?
- How could our model be combined with recent modeling studies on the role of extracellular spikes in high-frequency LFPs (Schomburg et al., 2012), to explain the detailed extracellular signature of SWRs?

New optogenetic, juxtacellular, pharmacological and imaging experiments (Lapray et al., 2012; Leão et al., 2012; Lovett-Barron et al., 2012; Royer et al., 2012; Pangalos et al., 2013) in addition to detailed computational biophysical modeling (Cutsuridis and Wenneckers, 2009; Cutsuridis et al., 2010a,b; Cutsuridis and Hasselmo, 2011; Cutsuridis et al., 2011; Taxidis et al., 2012, 2013), linking molecular, cellular and network phenomena to behavior, may bring light into these open questions and a better understanding of the memory consolidation process. With the advent of new and more advanced experimental techniques and the exponential increase in computational power, it is imperative for the experimental and computational communities to communicate with each other more closely, so as not to lose track of the bigger picture. Only then, they will be both successful in uncovering the biophysical mechanisms of SWR generation in the hippocampus and its relation to memory consolidation.

## Conflict of interest statement

The authors declare that the research was conducted in the absence of any commercial or financial relationships that could be construed as a potential conflict of interest.
